# let-7b and let-7c are determinants of intrinsic chemoresistance in renal cell carcinoma

**DOI:** 10.1186/s12957-015-0596-4

**Published:** 2015-05-08

**Authors:** Jingtao Peng, Ren Mo, Jian Ma, Jie Fan

**Affiliations:** Department of Urology, Shanghai First People’s Hospital, School of Medicine, Shanghai Jiaotong University, 100 Haining Road, Shanghai, 200080 People’s Republic of China; Department of Urology, Inner Mongolia Autonomous Region Peoples Hospital, 20 Zhaowuda Road, Hohhot, Inner Mongolia 010017 People’s Republic of China

**Keywords:** MicroRNAs, let-7b, let-7c, Renal cell carcinoma, 5-FU, Chemotherapy

## Abstract

**Background:**

Renal cell carcinoma (RCC) is characterized by inherent resistance to chemotherapy. Earlier studies demonstrated that microRNAs (miRNAs) might be involved in the chemosensitivity of cancers. MicroRNA let-7, a putative tumor suppressor, is dysregulated in many cancers. Our study aims to investigate the exact role of let-7 in chemotherapy sensitivity of 5-fluorouracil (5-FU) in RCC.

**Methods:**

The clinical significance of let-7b and let-7c expression in surgically resected specimens was assessed by qRT-PCR. Cell proliferation assay and colony formation assay were used to assess the survival of 786-O cells treated with let-7b or let-7c combined with 5-FU. Western blot was used to detect the expression of Akt2 and caspase-7. Luciferase assay was used to detect the direct binding of let-7b and let-7c to the 3′-untranslated region (UTR) of Akt2.

**Results:**

Expression of let-7b and let-7c was significantly decreased in 32 paired clear cell renal cell carcinoma tissue specimens and the dysregulation of let-7b was associated with pathological grade. Transfection of let-7b or let-7c combined with 5-FU inhibited proliferation and potentiated the antitumor efficacies of 5-FU at tolerated concentration. let-7b and let-7c suppressed the luciferase activity of reporter plasmid containing the 3′-UTR of Akt2. Overexpression of let-7b and let-7c reduced Akt2 expression, and Akt2 inhibition enhanced the sensitivity to 5-FU by affecting apoptotic pathway.

**Conclusions:**

Expression of let-7b and let-7c was frequently decreased in clear cell renal cell carcinoma tissues. The dysregulation of let-7b and let-7c may be involved in chemoresistance of RCC cells to 5-FU by down-regulating Akt2.

**Electronic supplementary material:**

The online version of this article (doi:10.1186/s12957-015-0596-4) contains supplementary material, which is available to authorized users.

## Background

Renal cell carcinoma (RCC) is the most lethal of the common urologic cancers and accounts for 2% to 3% of all adult malignant neoplasms. Approximately 30,000 new diagnoses of RCC are made each year in the United States, and 12,000 patients die of disease [1]. Surgical resection of the primary tumor for patients with localized disease remains the mainstay of therapy. However, a high proportion of patients with metastases at diagnosis or relapse following nephrectomy have a poor outcome due to its resistance to chemotherapy and radiotherapy, as it is widely accepted that RCC is among the most chemoresistant tumors [2]. The intrinsic drug resistance in RCC, always impairing the efficiency of chemotherapy, has been ascribed to the overexpression of multiple-drug resistance protein (P-glycoprotein) and multiple-drug resistance-associated protein 1 (MRP1) in 80% to 100% of RCC tumors [3]. Targeted therapies can offer significant clinical benefit, but rarely complete responses and development of resistance after a median of 5 to 11 months still remain a palliative treatment with a tremendous increase in the cost of care [4]. Traditional anticancer drug 5-fluorouracil (5-FU) is widely used in the treatment of a range of cancers. In RCC, rare response remains a significant limitation to the clinical use of 5-FU, although trials have indicated that responding patients survive longer [5]. Thus, it is of great significance to uncover the mechanisms of insensitivity of RCC to 5-FU and then find a way to re-sensitize RCC to anticancer drugs.

MicroRNAs (miRNAs) are approximately 22-nucleotide noncoding RNAs, generally involved in posttranscriptional gene regulation by binding to the 3′-untranslated region (UTR) of target mRNAs. Dysregulation of miRNAs occurs in various human cancers, and it is associated with the biological properties of cancers [6]. We have previously reported that miRNA let-7 acted as a tumor suppressor in RCC cell lines by down-regulating C-myc and C-myc target genes [7]. let-7 is also involved in the drug resistance. Enforced let-7 expression in hepatoma cancer sensitized cells to 5-FU by targeting cyclin A [8]. let-7 also affected sensitivity to tamoxifen treatment by targeting estrogen receptor-α signaling in breast cancer [9]. However, the role of let-7 in modulating the chemosensitivity of RCC is still largely unknown.

The serine/threonine kinase Akt plays a crucial role in cellular signaling pathway. In mammals, three isoforms of the Akt family have been identified, termed Akt1, Akt2, and Akt3, respectively [10]. AKT2 has been shown to be linked to aggression and drug resistance in a variety of human cancers, including ovarian cancer, breast cancer, endometrial cancer, and malignant gliomas [11-13]. Down-regulation of AKT2 sensitizes cancer cells to anticancer drugs by affecting apoptosis pathway such as caspases-3, caspases-6, caspases-9, PARP and p38 activity, and chemoresistance proteins such as MDR1 and MRP1 [11-13]. Akt2 has been implicated as the downstream of let-7 [14]. Therefore, we hypothesized that there is an association of Akt2 with let-7 in the resistance of RCC to anticancer drugs.

In the present study, we investigated the expression of let-7 in RCC tissues and found that the let-7b and let-7c were significantly down-regulated in renal tumors compared with normal tissues. We demonstrated for the first time that let-7b and let-7c could affect the sensitivity of RCC to 5-FU by targeting Akt2, which could reverse RCC’s intrinsic resistance to chemotherapy and enhance the effectiveness of anticancer drugs.

## Methods

### Clinical specimens from RCC patients

Thirty-two paired tissues were obtained from patients who underwent radical nephrectomy at the department of Urology, Shanghai First People’s Hospital, School of Medicine, Shanghai Jiaotong University from August 2012 to December 2013. The specimens were collected from normal region (outside the range of tumor at least 5 cm macroscopically) and tumor for each patient. All the specimens were stored at −80°C immediately after surgery until RNA extraction. The histological diagnosis was confirmed by examining hematoxylin and eosin (H & E)-stained original sections, simultaneously by two pathologists. Tumor stage was determined according to the American Joint Committee on Cancer (AJCC), and nuclear grading was classified according to the method described by Fuhrman et al. [15]. All patients were informed, and consent was given.

### Cell culture and transfection

The human RCC cell lines 786-O and 769-P were obtained from the American Type Culture Collection (ATCC) and cultured in RPMI 1640 medium (GIBCO, Grand Island, NY, USA) supplemented with 10% fetal bovine serum (GIBCO, Newcastle, Australia). The cells were incubated at 37°C in a humid atmosphere with 5% CO2. let-7b mimics, let-7c mimics, Akt2 siRNA, and the negative control were synthesized by Shanghai GenePharma Co. 786-O and 769-P cells seeded at 1 × 10^5^ cells/well in 6-well plates were transfected with let-7b or let-7c mimics or the negative control using Lipofectamine 2000 Transfection Reagent (Invitrogen, New York, NY, USA) at 50 nmol/l according to the manufacturer’s instruction. The information of microRNA and siRNA sequences is provided in Additional file 1.

### Cell proliferation analysis and colony formation assay

Cell viability was determined by CCK-8 (Dojindo Laboratories, Kumamoto, Japan) assay. 5-FU was purchased from QiLu Pharmaceutical (Jinan, China). 786-O cells were transfected with let-7b mimics, let-7c mimics, Akt2 siRNA, or negative control for 24 h before seeded into 96-well plates at 1,000 cells per well. For 5-FU treatment, 1-10^4^ μmol/l 5-FU was added into the growth medium after seeded overnight. At certain time points, the cells were incubated in CCK-8 in growth medium at 37°C for 4 h according to the manufacturer’s instruction. The absorbance in each well was measured at 450 nm using a microplate reader. For the colony formation assay, transfected 786-O cells were seeded into 6-well plates at 1,000 cells per well with growth medium containing different concentrations of 5-FU. The medium containing 5-FU was replaced every 3 days until the visible colonies formed. The colonies were fixed with 4% paraformaldehyde (Sigma-Aldrich Co., St. Louis, MO, USA) and stained with 0.1% crystal violet (Sigma-Aldrich Co., St. Louis, MO, USA). Colonies >50 μm in diameter were counted.

### Western blot analysis

The Western blot analysis was performed as described earlier [16]. Briefly, proteins were harvested for Western blot analysis 48 h after transfection. After denatured at 100°C for 5 min, samples were separated (30 μg per lane) on 10% SDS-PAGE (Bio-Rad Laboratories, Hercules, CA, USA) and then transferred onto a polyvinylidene difluoride membrane (Roche Applied Science, Mannheim, Germany). The membrane was blocked with 5% skim milk at room temperature for 1 h, incubated overnight at 4°C with Akt2 antibody (abcam, Cambridge, UK) and caspase-7 antibody (Cell Signaling Technology, MA), then incubated with anti-rabbit IgG secondary antibody (Cell Signaling Technology, MA). A β-Actin antibody was used to determine loading (Cell Signaling Technology, Danvers, MA, USA). Antibody binding was visualized by chemiluminescence (Boster Company, Wuhan, China).

### Luciferase assay

The Akt2 3′-UTR and control vectors were purchased from GeneCopoeia. 786-O cells were transiently transfected with Akt2 3′-UTR reporter plasmid or control vector and let-7b or let-7c mimics or negative control. Firefly luciferase activities were measured by using the Dual Luciferase Reporter assay system (Promega, Madison, WI, USA) 36 h after transfection. The results were normalized with *Renilla* luciferase.

### Total RNA extraction, reverse transcription, and qRT-PCR

Total RNA from tissues was extracted using the TRizol reagent (Invitrogen, Carslbad, CA, USA) as described previously [7]. Reverse transcription for miRNAs was performed using Universal cDNA Synthesis Kit (Exiqon, Vedbaek, Denmark) according to the manufacturer’s instruction. Primer sets for real-time PCR of let-7b-5p, let-7c-5p, and 5S rRNA were purchased from Exiqon. The SYBR Green Master Mix was from Applied Biosystems (Carlsbad, CA, USA). The ABI 7900 HT Sequence Detection System (Applied Biosystems, Carlsbad, CA, USA, and Life Technologies, Carslbad, CA, USA) was used for quantitative real-time PCR assay. 5S rRNA was used for normalization. The information of primer sequences is provided in Additional file 1.

### Statistical analysis

All statistical analyses were performed with SAS 8.0, and values were presented as mean ± SD from at least three separate experiments. The levels of let-7b and let-7c between the normal and RCC tissues were compared by Wilcoxon signed rank sum test. The relationship between let-7b and let-7c expression was analyzed by the Spearman rank correlation. Differences between the expression of let-7b or let-7c and clinicopathological features were assessed by *χ*^2^ test. Student’s *t*-test was used to evaluate the significance of inter-group differences. Values with *P* < 0.05 were considered statistically significant.

## Results

### Down-regulation of let-7b and let-7c in RCC tissues

To explore whether let-7 was dysregulated in RCC, qRT-PCR analysis was used to determine the expression levels of let-7 family (let-7a, let-7b, let-7c, let-7d, let-7e, let-7f and let-7 g) in 32 pairs of RCC and adjacent normal kidney tissues. The median age at diagnosis was 61 (range 40 to 93) years old. The clinical and pathologic characteristics of the patients were shown in Table 1.

**Table 1:** **Patient characteristics and clinicopathological factors by let-7 expression**

**Variables**	**Number of patients (%)**	**let-7b expression**			**let-7c expression**		
		**Low**	**High**	***P*** **value**	**Low**	**High**	***P*** **value**
Gender							
Male	24 (0.75)	19	5	0.3458	19	5	0.8050
Female	8 (0.25)	5	3		6	2	
Age(years)							
>65	12 (0.37)	9	3	1.0000	9	3	0.7405
≤65	20 (0.63)	15	5		16	4	
Tumor size(cm)							
>4	15 (0.47)	12	3	0.5395	10	5	0.1408
≤4	17 (0.53)	12	5		15	2	
Pathologic stage							
pT1 to T2	24 (0.75)	16	8	0.0593	17	7	0.0840
pT3 to T4	8 (0.25)	8	0		8	0	
Lymph node involvement							
N0	31 (0.97)	23	8	0.5575	24	7	0.5908
N1 to N2	1 (0.03)	1	0		1	0	
Grade							
I to II	23 (0.72)	15	8	0.0411	17	6	0.3569
III to IV	9 (0.28)	9	0		8	1	

**Figure 1:**
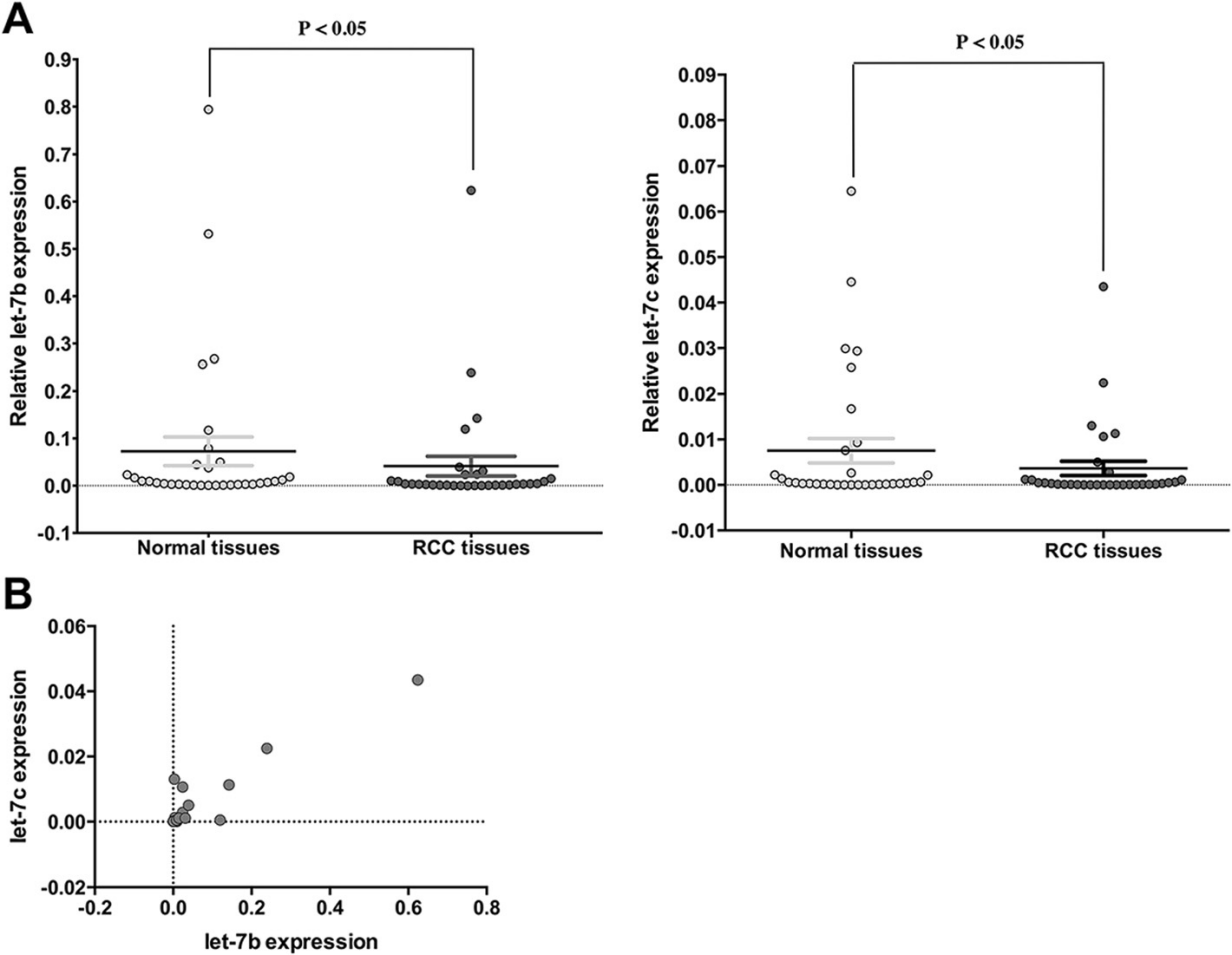
Expression of let-7b and let-7c in the 32 paired clinical specimens. **(A)** Expression of let-7b and let-7c was lower in 32 RCC clinical specimens than in 32 normal kidney specimens (*P* < 0.05). **(B)** let-7b and let-7c expression correlated positively (*r* = 0.810, *P* < 0.05).

In RCC tissues, let-7b and let-7c expression levels were the most significantly decreased compared to corresponding normal tissues (*P* < 0.05, Figure 1A). Thus, we chose these two miRNAs for further study. The Spearman rank test showed a positive correlation between let-7b and let-7c expression (*r* = 0.810, *P* < 0.05, Figure 1B). Then, we analyzed the data to see if any correlation exists between the expression of miRNAs and clinical characteristics. In patients with more advanced histopathological grade tumors, let-7b levels were significantly down-regulated (*P* < 0.05, Table 1).

### let-7b and let-7c enhanced the antitumor efficacies of 5-FU

**Figure 2:**
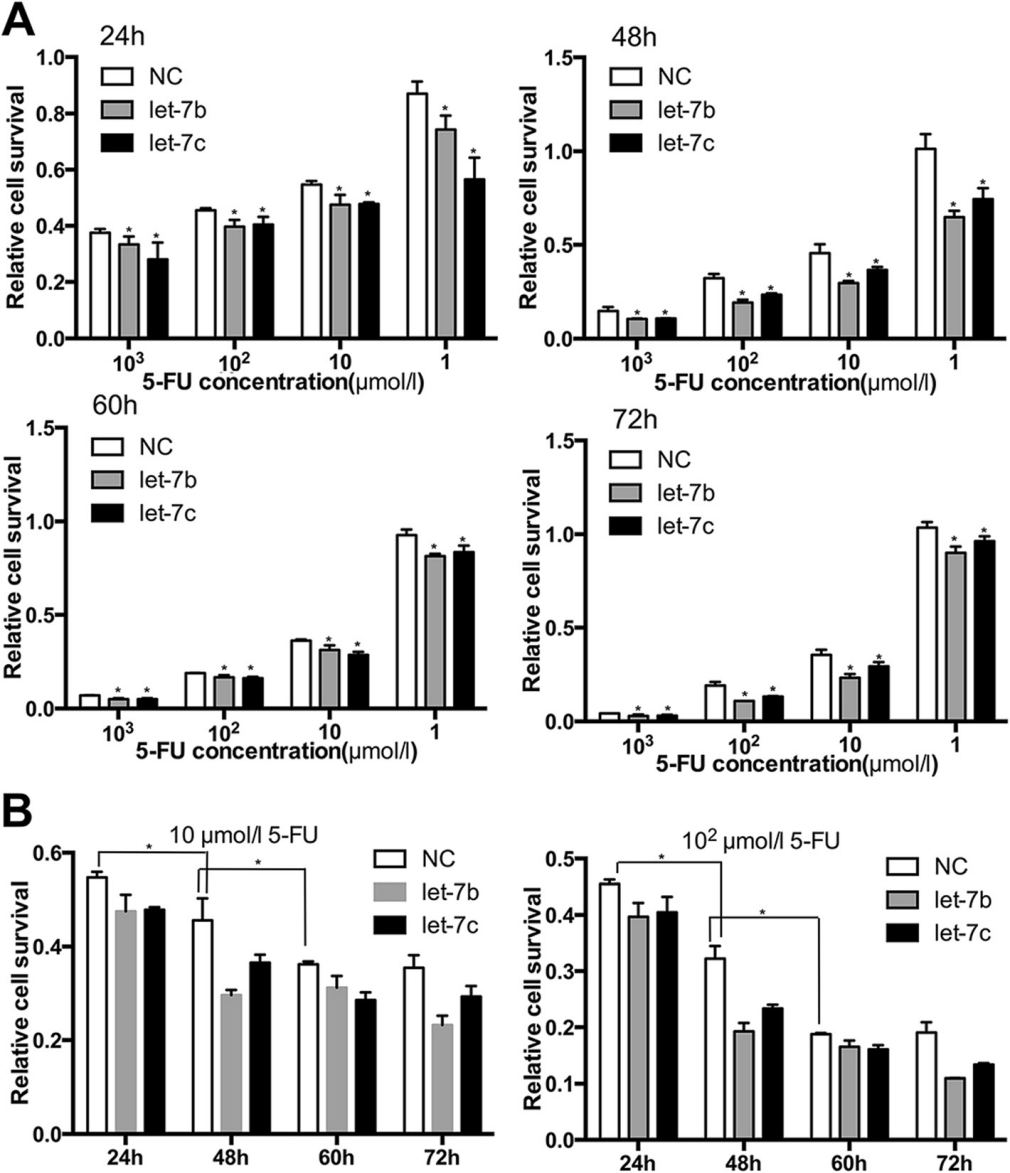
CCK-8 assay showed that let-7b and let-7c enhanced chemosensitivity of 786-O cells to 5-FU. **(A)** Relative cell survival of 786-O cells at certain time points (24, 48, 60, and 72 h) after transfected with let-7b or let-7c mimics and treated with 5-FU at progressive concentrations (1, 10, 10^2^, 10^3^, and 10^4^ μmol/l). **(B)** Relative cell survival of different groups with previous treatment for 24, 48, 60, and 72 h, in which 5-FU is at concentrations of 10 and 10^2^ μmol/l. Cell viability assay showed the inhibition of 786-O cells proliferation by transfection of let-7b and let-7c (*P < 0.05).

**Figure 3:**
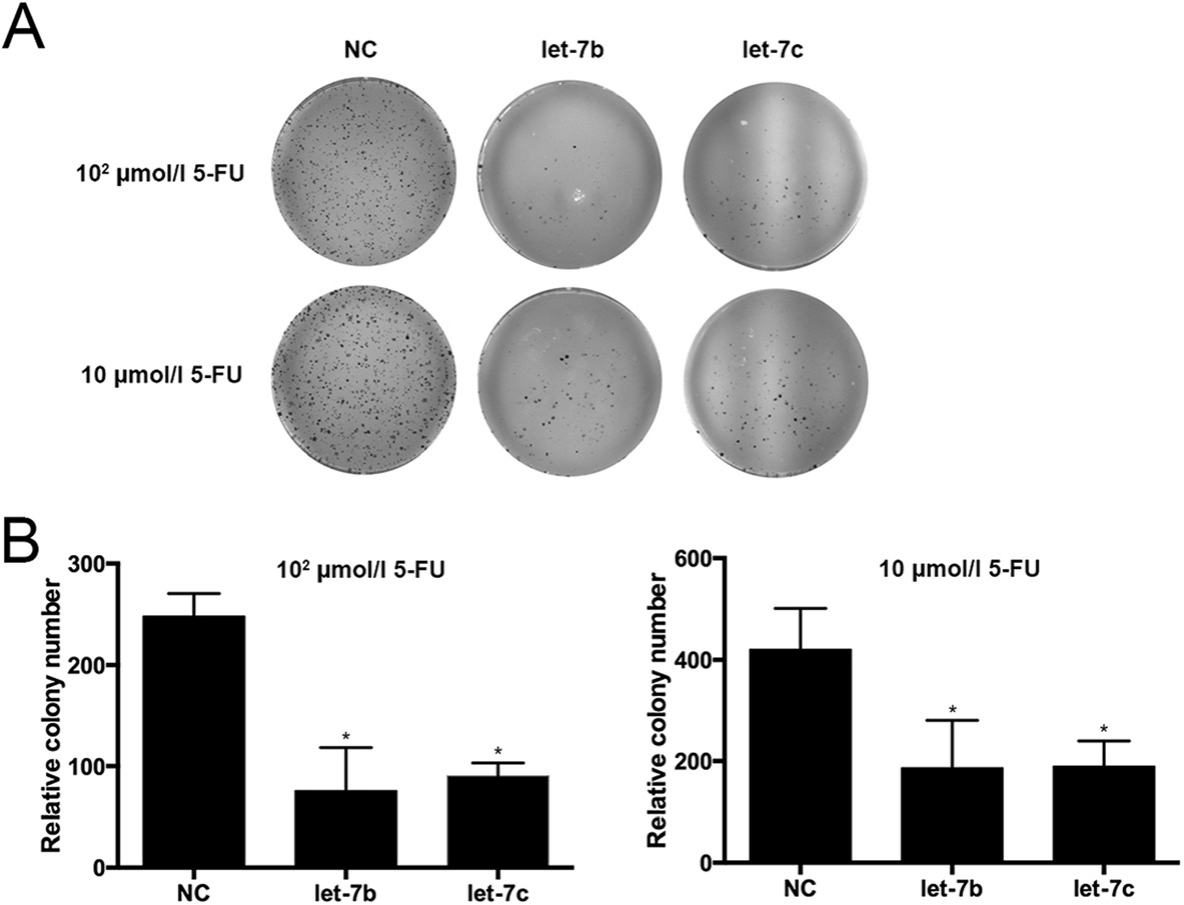
Colony formation assay was used to detect the cell growth activity. **(A)** Clones of transfected cells were less than control cells. The antitumor effects of 5-FU in transfected cells were more profound than those in the control cells. **(B)** Relative colony numbers of transfected cells and control cells. Less growth activity was found in transfected cells compared to control cells (*P < 0.05).

We previously noted that let-7 could inhibit proliferation of RCC cell lines [7]. We also wondered whether the inhibitory effect could be enhanced when combined with 5-FU treatment. For this purpose, let-7b or let-7c were transfected into 786-O cells, and then, the cells were treated with different doses of 5-FU. The CCK-8 assay showed that let-7b and let-7c transfected cells were more sensitive to 5-FU than control cells (*P* < 0.05, Figure 2). 5-FU monotherapy also showed antitumor effect, and the effect increased with the increasing concentrations. Similarly, colony formation assay showed that, in the condition of the same dose of 5-FU, transfected cells grew more slowly than control cells (*P* < 0.05, Figure 3). Thus, enforced expression of let- 7b and let-7c restored chemosensitivity to 5-FU in RCC cells, which suggests that co-treatment would reduce the dose of chemotherapy and reduce the side effect caused by 5-FU.

### Akt2 was a target of let-7b and let-7c

**Figure 4:**
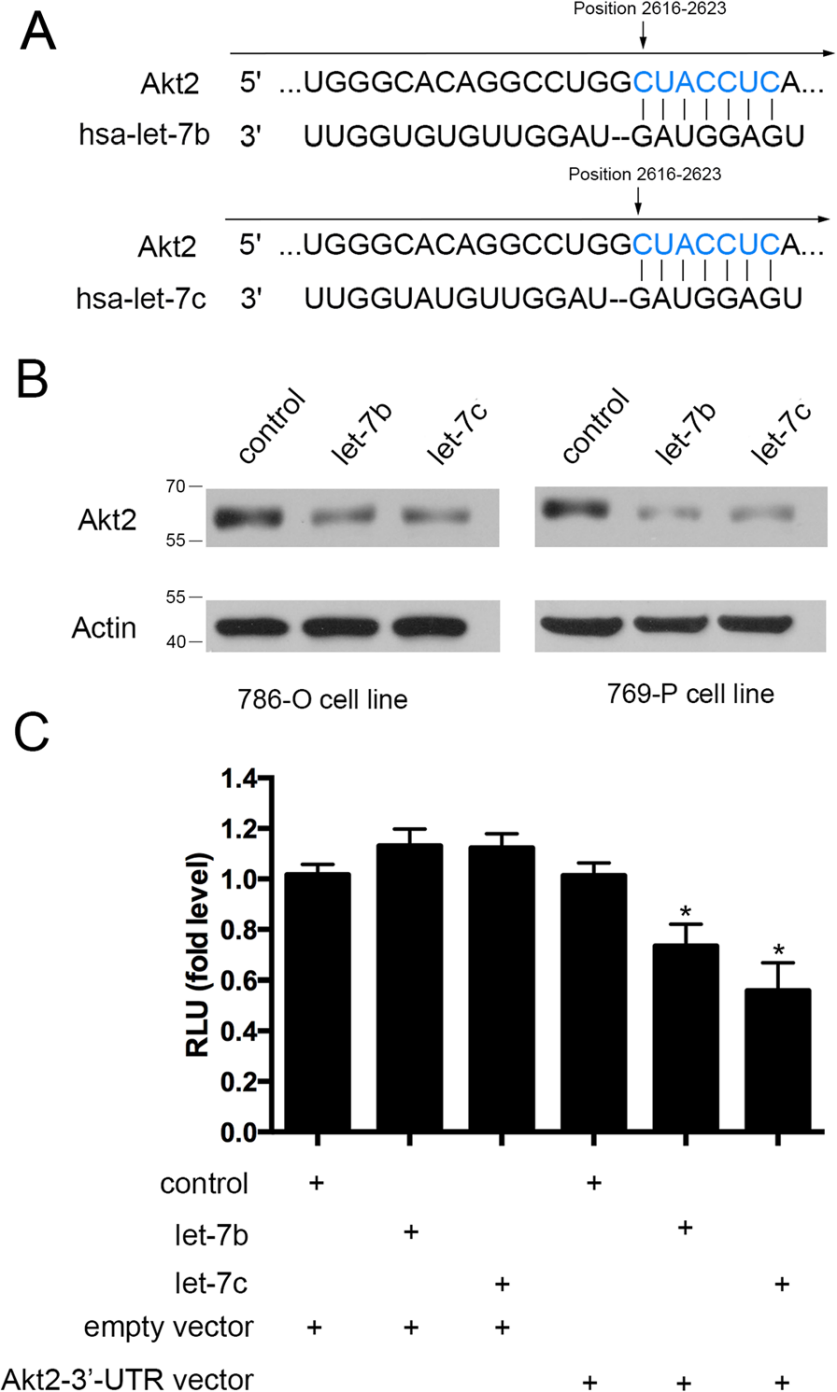
Akt2 is a functional target of let-7b and let-7c. **(A)** Schematic representation of Akt2 3′-UTRs as the putative target of let-7b and let-7c. **(B)** Western blot analysis demonstrated significant decreased expression of Akt2 in transfected cells. **(C)** Luciferase assay showed decreased reporter activity after co-transfection of Akt2 3′-UTR plasmid with let-7b or let-7c in 786-O cells (*P < 0.05).

To explore the mechanism of let-7b and let-7c mediated chemosensitivity, we hypothesized that let-7b and let-7c may inhibit proliferation of 5-FU-treated cells through down-regulation of Akt2. Firstly, Targetscan and miRBase database showed that Akt2 is a potential target of let-7 (Figure 4A), and also, Akt2 was a putative let-7 target in previous findings [14]. In addition, recent studies have shown that down-regulation of Akt2 expression appeared to sensitize cancer cells to typical chemotherapeutic agents [11-13]. We transfected 786-O and 769-P cells with let-7b and let-7c mimics or negative control. Forty-eight hours after transfection, Western blot analysis showed that the cells transfected with let-7b and let-7c mimics displayed decreased expression of Akt2 (Figure 4B). Furthermore, transfection of 786-O cells with Akt2 3′-UTR plasmid along with let-7b or let-7c led to a significant decrease in relative luciferase units when compared with empty vector or negative control (Figure 4C). These results indicated that Akt2 is the direct target of let-7b and let-7c in renal cancer.

### Akt2 inhibition enhanced the sensitivity to 5-FU through apoptotic pathway

**Figure 5:**
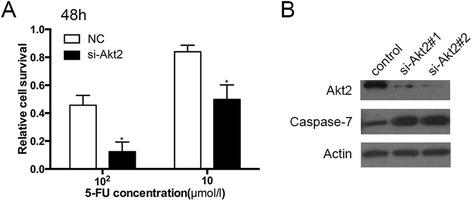
Knockdown of Akt2 enhanced the chemosensitivity through caspase-7. **(A)** Relative cell survival of 786-O cells 48 h after transfected with Akt2 siRNA and treated with 5-FU. Proliferation of 786-O cells after transfection of Akt2 siRNA was significantly reduced compared with negative control (*P < 0.05). **(B)** Akt2 and caspase-7 protein levels were assessed by Western blot in 786-O cells transfected with si-Akt2 (#1, #2) and negative control. Akt2 knockdown showed the increased expression of caspase-7.

We further explore mechanisms of potential reversal of chemoresistance through Akt inhibition. We initially tested 2 siRNAs to achieve 80% to 90% Akt2 gene knockdown and confirmed the results by Western blot (Figure 5B). Then, we selected one siRNA (si-Akt2#2) for further experiments. Inhibition of Akt2 expression by siRNA enhanced the sensitivity of renal cancer cells to 5-FU (Figure 5A). Next, we found the knockdown of Akt2 increased the expression of the pro-apoptotic factor caspase-7, indicating that Akt2 contributes to the chemoresistance through apoptotic pathway.

## Discussion

Surgery is thought to achieve cure in localized RCC, but for patients with recurrent or metastatic tumors, the efficacy of systemic therapy (chemotherapy, immunotherapy, and targeted therapy) is often limited. Although targeted therapy is highly recommended to improve survival, rarely complete responses and tremendous increase of the cost limit its wide application. 5-FU is an antimetabolite drug, which is the analogue of uracil with a fluorine atom at the C-5 position in place of hydrogen and enters cell as the same transport mechanism as uracil. The mechanism of anticancer effects of 5-FU has been ascribed to the inhibition of thymidylate synthase and incorporation of its metabolites into RNA and DNA. 5-FU is widely used in the treatment of a range of cancers, including colorectal, breast cancers, and head and neck cancers. 5-FU-based chemotherapy improves overall and disease-free survival of patients with above cancers [17]. However, RCC is insensitive to 5-FU, and it achieves response rates in the range of 5% to 8%. Clinically, 5-FU-based chemotherapy is considered moderately effective in RCC patients due to rare response and 5-FU-related severe toxicity [5,18]. Molecular mechanisms leading to 5-FU chemoresistance are poorly understood, thus factors that enhance the sensitivity of RCC cells to 5-FU may highlight predictive biomarkers or targets for therapy.

miRNAs have been proved to participate in the biological behavior of cancers as either tumor suppressors or oncogenes. Many studies, including our own, have reported that let-7 acts as a tumor suppressor in RCC [7,19]. Consistent with recently published reports that let-7 family was dysregulated in several types of human cancer, we found that let-7b and let-7c were down-regulated in RCC tissues compared with normal samples. Furthermore, our study revealed that decreased expression of let-7b was associated with grade in RCC, indicating that loss of this miRNA may be an early event in renal carcinogenesis and contribute to its intrinsic chemoresistance.

Chemoresistance represents a limiting factor for successful cancer therapy. It is of great benefit for optimizing cancer treatment to elucidate the mechanism of chemoresistance and identify the markers predicting the response to anticancer drugs. A few studies have reported the role of miRNAs in regulating sensitivity of cancer cells to chemotherapy. Boyerinas et al. [20] collected 20 patients with epithelial ovarian cancer who had received chemotherapy with Taxol and carboplatin and demonstrated that the expression of let-7d was significantly reduced in patients with recurrence after chemotherapy. Up-regulation of let-7g expression renders the cells more sensitive to treatment with either Taxol or vinblastine, by targeting IMP-1, resulting in destabilization of the mRNA of MDR1, which is a member of the adenosine triphosphate binding cassette transporters (ABC transporter family) that act by pumping drugs across the cell membrane to the extracellular space and known to affect treatment outcome by conferring intrinsic or acquired resistance to a variety of drugs. Another study by Sugimura et al. [21], which measured the expression of several miRNAs in biopsy samples from 98 patients with esophageal cancer who received preoperative chemotherapy, showed that low expression of let-7b and let-7c correlated significantly with poor response to chemotherapy, both clinically and histopathologically. *In vitro* assay showed that transfection of let-7c restored sensitivity to cisplatin through directly repressing interleukin (IL)-6/STAT3 pro-survival pathway. Here, we show that, besides the growth inhibition, let-7b and let-7c could also enhance the cytotoxicities of 5-FU to RCC, which is inherent resistant to anticancer drugs, and partially through down-regulation of Akt2.

Akt2 is a member of Akt family, which is an inactive cytosolic serine/threonine kinase activated by a phosphatidylinositol 3-kinase (PI3-K) in response to growth factors and plays a crucial role in cellular signaling pathway, regulating cell growth, differentiation, survival, and metabolism [10,22,23]. Previous studies indicated that Akt2 is associated with resistance to chemotherapy in a variety of malignancies. In breast cancer, Cheng et al. [24] demonstrated that Akt2 is transcriptionally up-regulated by twist leading to resistance to paclitaxel. Weng et al. [25] reported that Akt2 protects human ovarian cancer cells from docetaxel and paclitaxel-induced apoptosis via the p38 pathway and survivin expression. Girouard et al. [12] demonstrated that Akt2 down-regulation in endometrial cancer cells sensitizes cells to cisplatin by inducing the activation of pro-apoptotic factors such as the cleavage of caspases-3, −6, −9, and PARP. With regard to RCC, one recent study, which evaluated Akt activation by immunohistochemistry in 48 RCC biopsies, showed that elevated Akt activation could be a common finding and thus suggested that Akt might have an important role in the pathogenesis and progression of renal cell carcinoma [26]. Moreover, Sourbier et al. [27] found that inhibition of Akt phosphorylation induced cell apoptosis, thus indicating that activation of the Akt pathway in renal cell carcinoma might be one of the resistance mechanisms. Akt2 is thought to be a putative let-7 target in previous findings, but this is the first time to show that the association between let-7 and Akt2 plays an important role in the sensitivity to chemotherapy for RCC. Akt2 knockdown showed the same inhibition and increased expression of pro-apoptotic factor caspase-7, implying a functional interaction between Akt2 and the apoptotic pathway. These results suggest that treatment targeting this pathway is likely to enhance the sensitivity to chemotherapy. In addition, further research is needed to investigate whether the antitumor effect of other chemotherapeutic regimens could be enhanced by let-7 and the clinical use of let-7 combined with chemotherapy in patients with RCC.

## Conclusions

In conclusion, the results of the present study demonstrated that let-7b and let-7c were frequently down-regulated in RCC clinical specimens and dysregulation of let-7 may involve in chemoresistance of RCC cells to 5-FU. Moreover, the results also showed that the effect of let-7 expression on chemosensitivity is mediated though down-regulation of Akt2, further affecting pro-apoptotic factor caspase-7. This finding suggests that let-7 applied with tolerated concentration of 5-FU may be a potential therapeutic strategy for RCC therapy.

## Additional file

Additional file 1:
**The primers, microRNA mimics, and siRNA used in this study.**

## Abbreviations

5-FU: 5-fluorouracil
miRNAs: microRNAs
RCC: renal cell carcinoma
UTR: untranslated region
